# APE: A Command-Line Tool and API for Automated Workflow Composition

**DOI:** 10.1007/978-3-030-50436-6_34

**Published:** 2020-05-25

**Authors:** Vedran Kasalica, Anna-Lena Lamprecht

**Affiliations:** 8 University of Amsterdam, Amsterdam, The Netherlands; 9 University of Amsterdam, Amsterdam, The Netherlands; 10 University of Amsterdam, Amsterdam, The Netherlands; 11 University of Tennessee, Knoxville, TN USA; 12 University of Amsterdam, Amsterdam, The Netherlands; 13 Intellegibilis, Setúbal, Portugal; 14 Intellegibilis, Setúbal, Portugal; Department of Information and Computing Sciences, Utrecht University, 3584 CC Utrecht, The Netherlands

**Keywords:** Scientific workflows, Computational pipelines, Workflow management systems, Automated workflow composition, Workflow exploration

## Abstract

Automated workflow composition is bound to take the work with scientific workflows to the next level. On top of today’s comprehensive eScience infrastructure, it enables the automated generation of possible workflows for a given specification. However, functionality for automated workflow composition tends to be integrated with one of the many available workflow management systems, and is thus difficult or impossible to apply in other environments. Therefore we have developed APE (the Automated Pipeline Explorer) as a command-line tool and API for automated composition of scientific workflows. APE is easily configured to a new application domain by providing it with a domain ontology and semantically annotated tools. It can then be used to synthesize purpose-specific workflows based on a specification of the available workflow inputs, desired outputs and possibly additional constraints. The workflows can further be transformed into executable implementations and/or exported into standard workflow formats. In this paper we describe APE v1.0 and discuss lessons learned from applications in bioinformatics and geosciences.

## Introduction

Computational pipelines, or workflows, are central to contemporary computational science [5]. The international eScience community has created a comprehensive infrastructure of tools, services and platforms that support the work with scientific workflows. Numerous scientific workflow management systems exist [1, 29], some of the currently most popular being Galaxy [10], KNIME [6] and Nextflow [7]. While these systems free their users from many technicalities that they would have to deal with when conventionally programming workflows, the identification of suitable computational components and their composition into executable workflows remains a manual task.

The idea of *automated workflow composition* is to let an algorithm perform this process. Based on a loose specification of the intended workflow (for example in terms of available workflow inputs and desired outputs, or principal steps to take), it would automatically generate suitable, executable workflows. It has been shown that program synthesis [11] and AI planning techniques [8] can be used to implement such functionality [20, 22, 23]. Some workflow management systems, such as jORCA/Magallanes [15], jABC/PROPHETS [21, 24] and WINGS [9], provide automated workflow composition functionality based on such techniques. However, the tight integration with the respective workflow systems makes it difficult or even impossible to use this functionality in other environments.

Therefore we have developed APE^1^ (the Automated Pipeline Explorer) as a command-line tool and API for automated workflow composition. It is designed to be independent from any concrete workflow system, and thus ready to be used in other workflow management systems, tool repositories or workflow sharing platforms as needed. Internally, APE uses a SAT-based implementation of a temporal-logic process synthesis method, inspired by the approach behind the PROPHETS framework [21, 27] and described in detail [17]. In a nutshell, the framework uses an extension of the well known Linear Temporal Logic (LTL) to encode the workflow specification. This specification is translated into a propositional logic formula that can be processed by an off-the-shelf SAT solver, with the resulting solutions representing possible workflows for the specification.

In this paper, we introduce APE v1.0 from an application point of view. Section 2 describes how to set it up for use by providing a semantic domain model. Section 3 focuses on the automated composition of workflows based on the domain model and custom workflow specifications. Section 4 describes how APE-composed workflows can further be transformed into executable implementations and/or exported into standard workflow formats. Section 5 discusses lessons learned from applications of APE in bioinformatics and geosciences. Section 6 concludes the paper.

## Domain Model

The semantic domain model constitutes the knowledge base on which APE relies for the automated composition of workflows. It comprises a domain ontology and a collection of semantically annotated tools. The domain ontology provides taxonomic classifications of the data types and operations in the application domain, as a controlled vocabulary of technical terms. Tools in the domain model are semantically annotated with their inputs, outputs and operations, using terms from the ontology. Additionally, the domain model might include (temporal-logic) constraints to express further domain knowledge or rules.

**Fig. 1.**
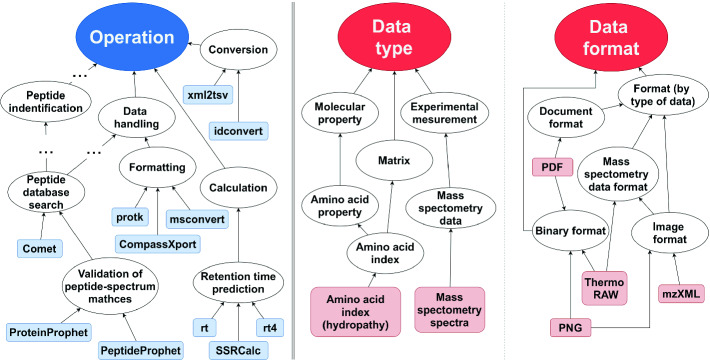
Fragment of a bioinformatics domain ontology.

**Table 1.** Fragment of an annotated set of bioinformatics tools [14].

Name	Operation	Data input (type/format)	Data output (type/format)
Comet	Peptide database search	**Mass spectrum**	**Peptide identification**
		**mzML** or **mzXML**	**pepXML**
msconvert	Formatting Filtering	**Mass spectrum**	**Mass spectrum**
		**MGF** or **mzXML** or **mzML**	**MGF** or **mzXML** or **mzML**
Peptide Prophet	Peptide identification Statistical modelling	**Peptide identification**	**Peptide identification**
		**pepXML** or **mzIdentML**	**pepXML**
rt4	Retention time prediction	**Peptide property**	**Amino acid index (hydropathy)**
		**TSV** or **pepXML**	**TSV **or **XML**
xml2tsv	Conversion	**Peptide identification**	**Peptide identification**
		**mzIdentML**	**TSV**
SSRCalc	Retention time prediction	**Peptide property**	**Amino acid index (hydropathy)**
		**Textual format** or **TSV**	**Textual format**
**...**			

For example, Fig. 1 and Table 1 show fragments of a bioinformatics domain model from a recent case study on automated workflow composition in proteomics [25]. The domain ontology (see Fig. 1) was directly derived from the popular bioinformatics data and methods ontology EDAM [12]. Table 1 shows a few tool annotations from the same case study. Each tool is semantically annotated with the operation(s) it performs and its input and output data types and formats, using terms from the respective taxonomies. These annotations were directly derived from the bio.tools registry [13, 14], a large collection of EDAM-annotated bioinformatics tools. Note that in this example, two dimensions (type and format) are used for the annotation of the input and output data. Other applications need only one (e.g. format), and yet others have more than two required dimensions. Hence, APE supports the use of multiple disjoint taxonomy trees to represent the required dimensions of data characterization.

Technically, we rely on existing and (de facto) standard formalisms for the representation of the domain model. APE loads the domain ontology from a file in Web Ontology Language (OWL) format. The tool annotations are represented in JavaScript Object Notation (JSON) format, following the schema that is used in the bio.tools registry [2].

## Automated Workflow Composition

**Fig. 2.**
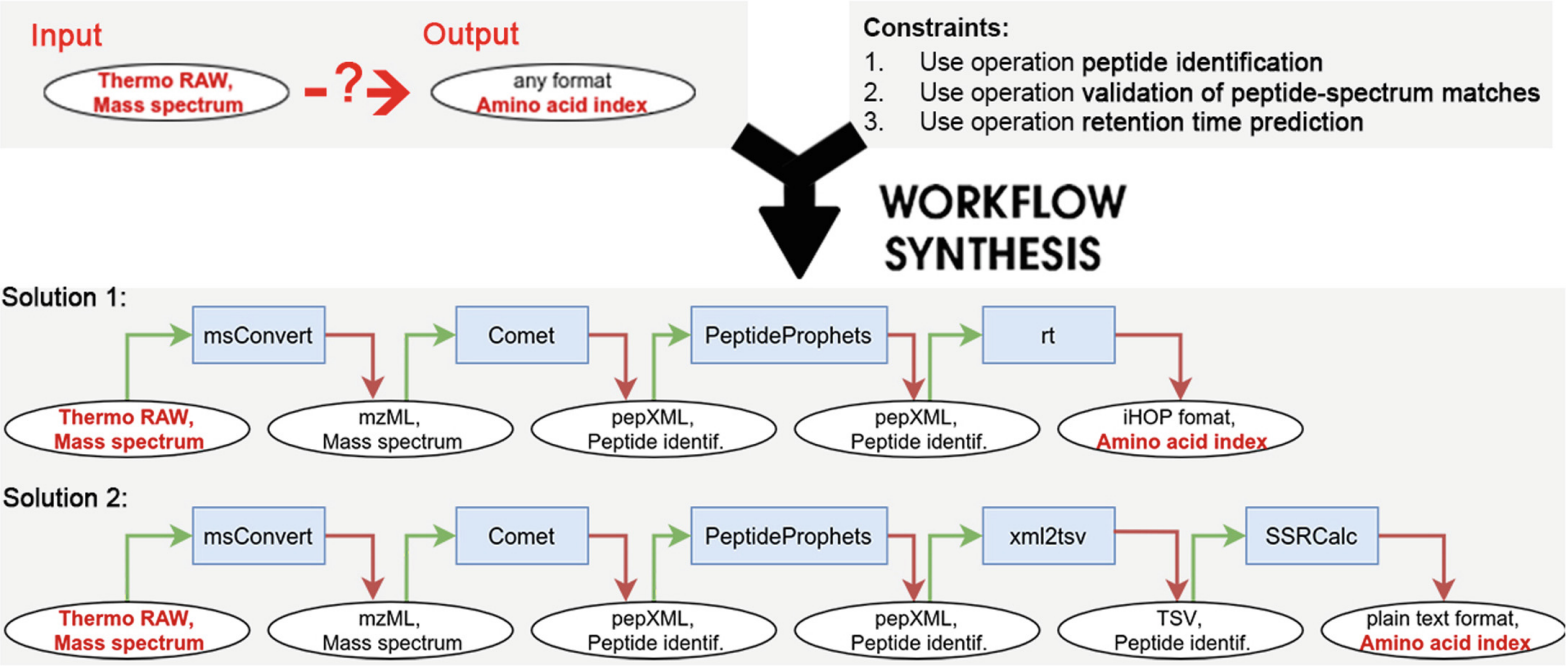
Automated composition of a proteomics workflow.

Once the domain model has been configured, APE is ready to be used for automated workflow composition. Therefor the user specifies the workflow inputs, intended outputs and additional constraints that the workflow has to fulfill. Internally the constraints are expressed in a formal (temporal) logic, but the APE interfaces expose them in the form of intuitive natural-language templates. For example (as illustrated in Fig. 2), one workflow specification from the proteomics case study consists of “Mass spectrum” type in “Thermo RAW format” as input, “Amino acid index (hydropathy)” (in any format) as output, and constraints specifying to use tools that perform the operations “peptide identification”, “validation of peptide spectrum matches” and “retention time prediction” (constraint template “Use operation *X*”). These operations are abstract terms from the ontology, known to scientists from the domain. This shows that formulating such constraints does not require knowledge of all available tools that fit the description. Based on the given specification APE synthesizes workflows that fulfill the specification by construction. Figure 2 shows two of many possible workflow solutions for the example specification.

Automated workflow composition with APE can be performed through its command line interface (CLI) or its application programming interface (API). While the CLI provides a simple means to interact and experiment with the system, the API provides more flexibility and control over the synthesis process. It can also be used to integrate APE’s functionality into other systems.

### Command Line Interface (CLI)

When running APE-<version>.jar from the command line, it requires a configuration file as a parameter and executes the complete automated workflow composition process accordingly. This JSON-based configuration file provides references to all therefor required information:

1. The domain model (as described in Sect. 2), provided as a pair of a well-formatted OWL and JSON files,
2. the workflow specification, provided as a list of workflow inputs/outputs and template-based workflow constraints, and
3. parameters for the synthesis execution, such as the number of desired solutions, output directory, system configurations, etc.

APE then writes the synthesized workflows into the defined output directory. Each solution consists of a text file that describes the steps of the workflow, a graphical representation, and a shell script that implements the workflow (depending on the availability of suitable shell commands in the tool annotations).

### Application Programming Interface (API)

Like the CLI, the APE API relies on a configuration file that references the domain ontology, tool annotations, workflow specification and execution parameters. However, the API allows to edit this file programmatically, and thus for instance add constraints or change execution parameters dynamically. This is useful, for instance, for providing more interactive user interfaces or for systematically exploring and evaluating workflow synthesis results for varying specifications and execution parameters.
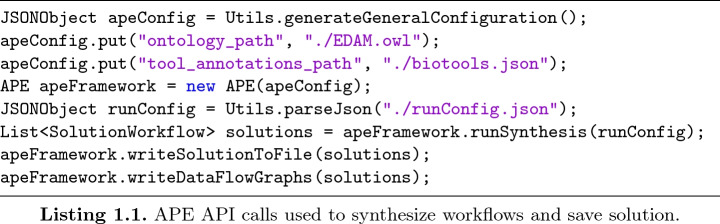


Listing 1.1 shows a small example of using the APE API for synthesizing a set of workflows similar to the example in Fig. 2. First, the paths to the domain ontology and tool annotation files are added to the APE configuration object. Then a new instance of the APE framework is created based on the configuration, and the workflow synthesis algorithm is executed with the provided run configuration. The result of the synthesis run is a list of solutions obtained from the SAT solver, which are written into the output directory in textual and graphical (data-flow) format.

**Fig. 3.**
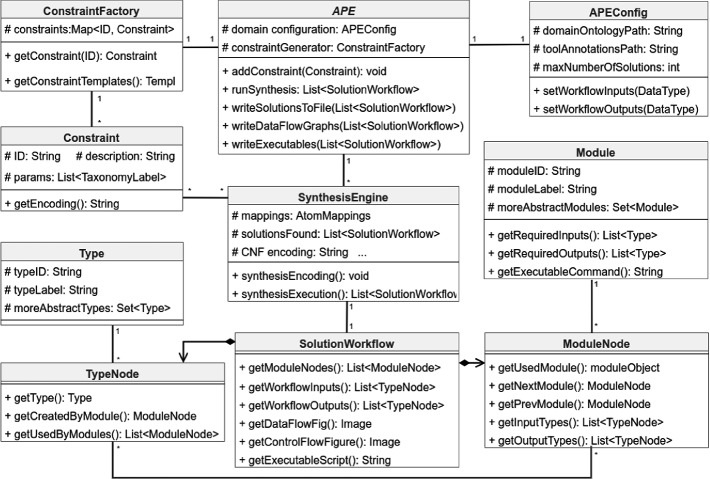
Fragment of the APE API.

The APE API provides further functionality, allowing for a more fine-grained interaction with the APE framework. Figure 3 outlines the API, for brevity focusing on the most relevant fields and functions. The *ConstraintFactory* and *Constraint* classes allow for the retrieval of constraint templates and for adding new or removing existing constraints, thus further constraining or loosening the specification, respectively. As shown in the example code above, the *APE* class constitutes the main interface for interaction with the framework. It is used to define the execution parameters as well as the output formats. Once the library has generated the solutions, they are provided as a list of *SolutionWorkflows*. Each solution is represented as a directed graph that comprises type and tool nodes (internally named modules). The interface for working with the workflow solutions (further elaborated in the next section) is provided by the classes *SolutionWorkflow*, *TypeNode* (representing type instances) and *ModuleNode* (representing tool instances).

## Workflow Implementation

As mentioned above, APE provides functionality for exporting the synthesized workflows as textual representations, in the form of (data-flow and control-flow) graphs and as executable shell scripts. In practice it is often desirable to implement workflows in one of the languages used by popular workflow management systems, in order to be able to execute them with the respective workflow engines. Given the large number of existing workflow languages, it is however not feasible for APE to provide ready-to-use export functionality for all of them. Instead, the information contained in APE’s own workflow representation can be used to create workflows in other languages. In the following we describe the APE workflow format and demonstrate how the contained information can be used to create corresponding workflows in the Common Workflow Language (CWL) [4]. This feature is going to be integrated to the APE API in the near future. The mapping process described in this paper can furthermore serve as a template for the translation of APE results to other workflow formats, such as NextFlow [7], SnakeMake [19] or the Workflow Description Language (WDL) [3].

**Fig. 4.**
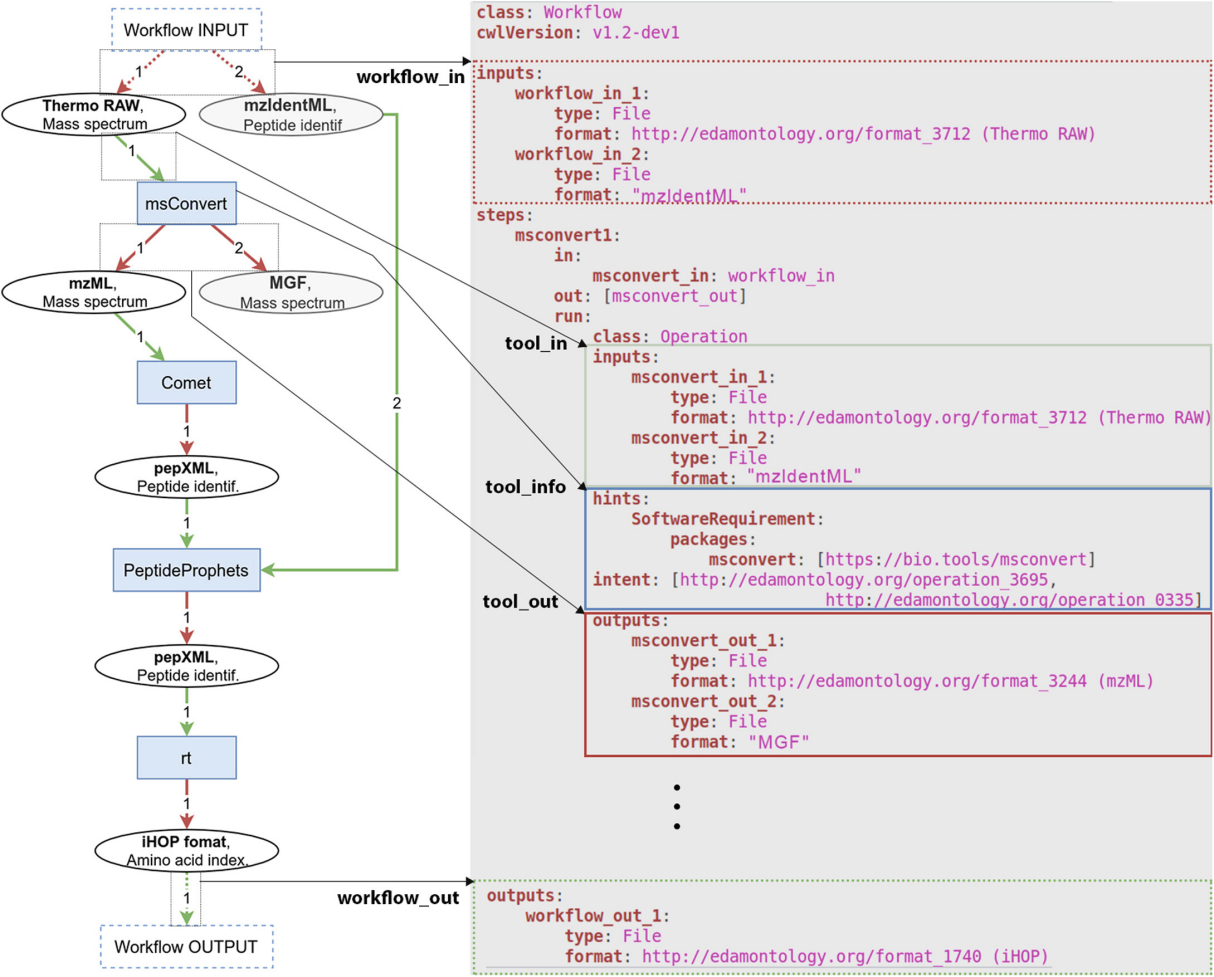
Workflow in APE’s native format (left) and corresponding CWL (right).

### APE Workflow Format

APE represents the workflow solutions in the form of directed graphs. The left-hand side of Fig. 4 shows an example. Nodes in the graph represent instances of data (depicted as ellipses) and executions of operations (rectangles), while the edges represent inputs and outputs of these tools, shown as green and red arrows, respectively. In addition, labels on the edges represent the order in which they are given as arguments to the tools. This graph provides the trace information that is needed to create the workflow in another language.

The APE API provides a set of functions to aid the interaction with the graph structure (see class *SolutionWorkflow* in Fig. 3). The workflow inputs can simply be retrieved using the corresponding function of the *SolutionWorkflow* class, which returns it as a list of *TypeNodes*. Generally, each *TypeNode* comprises a (possibly empty) tool node that generated it as an output, a (possibly empty) list of tools that used it as an input, and a concrete data *Type* that identifies it. Further, the *SolutionWorkflow* class provides a function for retrieving the tools used in the workflow as list of *ModuleNodes* (sorted according to their order of execution), making it easy to iterate over all tools used in the workflow. Each *ModuleNode* provides information about the next and the previous *ModuleNode* in the sequence, the *TypeNodes* used as inputs and generated as outputs by the tool, as well as information about the actual tool (executable script, see class *Module*) that provides the information needed for its execution. Finally, the workflow outputs are provided in the same format as the initial inputs. Note that for this example the first proposed solution from Fig. 3 was artificially extended with additional inputs and outputs (depicted as gray ellipses) for illustrative purposes.

### Translation to CWL

The Common Workflow Language^2^ (CWL) [4] has recently emerged as an open standard for describing scientific workflows across platforms. It is increasingly adopted by the scientific community, with CWL support being added to popular scientific workflow management systems like, for example, Galaxy [10] and Toil [28]. CWL is a declarative language that focuses on workflows composed from command line tools. Basically, it describes a set of steps and dependencies between those steps. CWL has its roots in “make” and similar tools, and like them it determines the order of execution based on these dependencies between tasks, i.e. if there is a required order of the operations or if they can even be executed concurrently. Conveniently, the main CWL structure is quite similar to the APE workflow structure. A basic workflow (see right-hand side of Fig. 4) comprises a configuration header, a list of workflow inputs, steps to be performed and workflow outputs. The input/output dependencies have to be explicitly defined, again in line with our data trace workflow representation. The tools in CWL usually include a command field, explicitly defining the corresponding command line operation. In addition, they can be configured to run tools from Docker containers automatically, allowing for more flexible and scalable workflow implementations.

However, as the fully automatic configuration for execution is not always feasible, the upcoming CWL version 1.2 will introduce *abstract workflows*. These workflows use descriptive containers instead of directly executable operations, and require additional (manual) configuration to become executable. The abstract containers are represented using the *intent* label (see Fig. 4). Given that functional description of tools is sufficient for workflow discovery with APE, the abstract CWL workflows match well with APE’s own workflow representation. Furthermore, the bio.tools registry used as source for the tool annotations in the aforementioned bioinformatics case study is a typical example of such a set of tools. The repository contains the semantic annotations of the tools, but still might require some additional work from the user in order to execute the tool itself. Hence APE discovers workflows composed of tools that are not necessarily available on the local system, potentially requiring the installation and configuration of the tools on the execution system first.

To translate and APE workflow into CWL format, it is sufficient to 1) describe the original inputs, 2) iterate through the tools in the workflow sequence and specify the inputs used and outputs generated, and finally 3) specify the workflow output list. The right-hand side of Fig. 4 shows the CWL representation of the APE workflow on the left. To create it, first, the list of input objects is translated into a list of inputs that are annotated using their formats (see Label **workflow_in**). This means that some information about the data get lost in the translation (specifically the type description). However, as at runtime the format is sufficient to perform the execution, this is not a problem. Second, each tool in the sequence is described. The description involves a definition of the inputs, outputs and tool execution specification (mappings are annotated using labels **tool_in**, **tool_out** and **tool_info**, respectively). The most important part of the step is to keep track of the exact source of the tool inputs as well as to provide sufficient tool description that would allow for its execution. The input information is already part of the formalism, as APE keeps track of data flow traces for each data instance. The only requirement is to properly use the identifiers provided when creating the mappings to CWL. Regarding the tool descriptions, as long as the provided tool annotation file contains sufficient information, it can be translated into CWL. Third, the final workflow outputs need to be specified based on the given solution description (see Label **workflow_out**).

## Applications and Lessons Learned

The development of APE was accompanied by three concrete application scenarios for automated workflow composition: 1) The proteomics case study mentioned earlier in this paper [25], 2) a case study on cartographic map generation [16], and 3) geospatial data transformations in the QuAnGIS project [18, 26]. The experiences from these applications, in particular the feedback from the involved domain experts, influenced the design decisions that we took during the development of the APE CLI and API. While initial versions of all three application scenarios have been created with PROPHETS, they have meanwhile been migrated to APE completely and are publicly available^3^.

Naturally, the quality of the workflows obtained through APE essentially depends on the quality of the semantic domain model (ontologies and functional tool annotations). Hence it is crucial to involve domain experts in the domain modeling process, or to rely on sources that have been created by expert communities, such as the EDAM ontology and bio.tools registry that we use in bioinformatics applications of APE. Essentially, the idea is that the domain model is provided and maintained by a small group of domain experts, and used by a larger and broader audience to automatically compose workflows. As a positive side effect on domain modeling, using APE for the systematic generation and evaluation of workflows from varying specifications proved to be helpful to revise and improve ontologies and annotations.

Initially we used a tabular format for the tool annotations, like the one shown in Table 1, because spreadsheets are easy to discuss with collaborators, and the corresponding CSV files easy to process programmatically. However, this approach quickly turned out to be insufficient to adequately capture non-trivial tool annotations. In the proteomics case study, we annotated tools’ inputs and outputs with both data type and format terms from EDAM. As the tools have varying numbers of inputs and outputs, however, they could not be properly annotated in the tabular format with a fixed number of columns. To increase the expressiveness of APE’s tool annotation template, but at the same time reuse an existing formalism, we decided to adopt the JSON-based tool annotation schema used in the bio.tools registry [2], which includes a well-defined and flexible mechanism for functional tool annotation. This has of course extremely simplified the setup of bioinformatics domain models based on bio.tools, but it has also shown to be easy to use in the other application domains.

The APE CLI and API aim to be easy-to-use, but clearly target a tech-savvy audience with a certain level coding and/or scripting confidence. To reach a broader audience, an intuitive interface that can be used without technical experience or specific training is required. As a proof of principle, we recently developed Burke (a Bio-tools and edam User interface foR automated worKflow Exploration^4^). Preconfigured to the domain model of the proteomics case study, it provides the automated workflow composition functionality of APE through a browser-based graphical interface. Users can select input and output data types and formats, as well as constraint templates and their instantiations, from drop-down menus that are filled with the relevant EDAM terms. They can configure and run APE’s synthesizer from the interface, and subsequently inspect the results, which are presented in a convenient tabular format. Feedback on Burke by APE novices has been very positive, hence we plan to develop a more sophisticated web interface for APE in the scope of future work on the framework.

A graphical interface has also the potential to overcome another limitation of the framework: Currently it is a tedious process to compare the different possible workflows generated by APE. This is however needed to make an informed decision about which of the potentially many possible workflows to select for implementation and execution. A graphical interface provides more possibilities for dynamically filtering, aggregating and displaying workflow candidates according to different criteria. Which criteria would actually provide meaningful information for workflow selection is currently an open question. This is another challenge that we are going to work on in the future.

## Conclusion

We believe that automated workflow composition will take the work with scientific workflows to the next level. On top of today’s comprehensive eScience infrastructure, it enables the automated generation of possible workflows for a given specification. In this paper we introduced APE v1.0 (the Automatic Pipeline Explorer), a command line tool and API that automates the exploration of scientific workflows. APE is under active development and continuously improving through the experiences and feedback from applications.

Future work on the APE framework will address different remaining challenges of usability and scalability. We are going to work on more end user-oriented interfaces that support better the whole life cycle of specifying, synthesizing, comparing, selecting, implementing and benchmarking computational pipelines. With growing domain models, the runtime performance of the underlying synthesis algorithm is likely to become a bottleneck. We have started to work on domain-specific search heuristics to improve synthesis performance and allow the approach to scale.

## Footnotes

1 https://github.com/sanctuuary/ape.

2 https://www.commonwl.org/.

3 https://github.com/sanctuuary/APE_UseCases.

4 https://github.com/sanctuuary/Burke_Docker.
